# Changes in Body Mass and Composition of the Body as Well as Physical Activity and Time Spent in Front of the Monitor by Students of the Wroclaw University of Health and Sport Sciences during the Period of COVID-19 Restrictions

**DOI:** 10.3390/ijerph18157801

**Published:** 2021-07-23

**Authors:** Agnieszka Chwałczyńska, Waldemar Andrzejewski

**Affiliations:** 1 Department of Human Biology, Faculty of Physiotherapy, Wroclaw University of Health and Sport Sciences, I.J. Paderewskiego Av. 35, 51-612 Wrocław, Poland; 2 Faculty of Physiotherapy, Wroclaw University of Health and Sport Sciences, I.J. Paderewskiego Av. 35, 51-612 Wrocław, Poland; waldemar.andrzejewski@awf.wroc.pl

**Keywords:** body composition, COVID-19 restrictions, physical activity after the pandemic, weight and composition of the body after the pandemic

## Abstract

The aim of the study is to assess the impact of changes in daily physical activity during the blockade (March 2020–February 2021) on the mass and segmental composition of the body of young people. Material and research methods: In total, 120 people from the sports and medical university aged 19.8 (±0.8) years, average height 173.2 (±9.2) cm, body mass 66.1 (±12.8), and BMI 19.2 (±5.9) kg/m^2^. The research was carried out in two stages. The total and segmental body mass of the respondents and the change in physical activity during the pandemic were assessed twice (December 2019, February 2021). There was a statistically significant increase in body mass in men, an increase in total fat mass in women, and statistically significant changes in the distribution of fat mass in both sexes. Conclusions: In the studied group, there was a change in the forms of physical activity from strength and group activity to endurance activity (running forms, cycling.) and individual activity. The subjects showed a statistically significant increase in body fat, regardless of gender, and in the upper limbs in men.

## 1. Introduction

Since March 2020, daily functioning in Poland was to an extent limited due to the implementation of various restrictions such as interpersonal contact, trade, leisure, sports activities and face to face teaching. The current situation (March 2020–April 2021) is atypical in all respects. The restrictions introduced by the Polish government consisted mainly in restrictions on activity outside the place of residence. Universities were closed—by introducing distance learning; sports centers were closed—indoor fitness clubs, outdoor gyms, swimming pools, sports fields, a ban on team games outside of Polish representatives was introduced. An important element of the blockade was the closure of restaurants and pubs, the cancellation of concerts, and the closure of cultural centers (including cinemas, theaters). In the period March–June 2020 and October 2020–February 2021, the possibility of meeting in a group of more than five people was also limited [1,2]

One of the aspects most influenced by the present situation is the ability to perform physical activity and consequently, fulfilling the standards recommended in this regard by WHO. The relationship between physical activity and a healthy body weight has been known for a long time. In order to maintain a healthy body weight, an adult should undertake physical activity of at least 150–300 min a week of moderate intensity, and at least twice a week, exercise to strengthen the muscles of moderate or greater intensity. According to the latest WHO guidelines (2020), the aspect of life that affects its quality is not only physical activity, but also a sedentary lifestyle. According to this study, a sedentary lifestyle in adults is associated with adverse health effects such as increased incidence of cardiovascular diseases, cancer, type 2 diabetes, and increased mortality due to lifestyle diseases. Therefore, the latest WHO guidelines, in addition to the recommendations for physical activity, pay great attention to the need to limit sedentary lifestyle and replace it with even low-intensity activity [3]

Limiting physical activity affects the quality of life, as well as health. Adequate physical activity not only influences one’s mental health, but also helps maintain a correct body mass [4,5,6]. This plays a vital role in the preservation of health. Changes in physical activity dictated by the pandemic are still not fully known, as the problem of the spread of the SARS-CoV-2 virus has not yet been resolved, hence, we have not yet returned to how things were prior to the outbreak [7,8,9]. Many authors have observed a strong correlation between excessive body mass and susceptibility of being infected by the SARS-CoV-2 virus, as well as a link between increased body mass and severity of symptoms and mortality rate [10,11,12,13,14,15]. Nonetheless, there are relatively few studies outlining the effects of restricted mobility during lockdown on physical activity and body composition. Romero-Blanco et al. in a study conducted on a group of students of health sciences, observed an increase in physical activity in people with a normative body weight with a simultaneous increase in the amount of time spent in a sitting position [16]. An increase in sedentary lifestyle, especially in front of the monitor, was also observed by research in Italian, Spanish, German and Australian students, while physical activity significantly decreased in these groups [17,18,19,20,21,22,23]. On the other hand, in the systematic review of Stocwell et al. including students in the group of adults, an increase in physical activity was observed resulting from increased free time (remote work at home gave greater opportunities for individual exercises) with a simultaneous increase in time spent in front of the monitor [24].

The restrictions introduced due to the pandemic affected all spheres of everyday life—economy, education, social life, especially interpersonal contacts, employment and many others [16,17,18,19,20,21,22,23,25]. The available literature highlights, inter alia, the increase in indicators of mental stress in women in the US compared to 2018 [25] or mental anxiety in people over 16 in the UK [26], which do not result from typical changes observed in previous years. The authors of these publications indicate as one of the reasons for this situation the limitations that were introduced in the studied countries due to the COVID-19 pandemic. Pierce et al. observed in their research that the group with the greatest increase in mental anxiety includes young women, people studying or having an insecure job [27].

One of the elements influencing body mass and composition is diet. Romero-Blanco, et al. in studies conducted on a group of students, observed that physical activity decreased among the respondents following the Mediterranean diet, while it increased in those who did not follow the Mediterranean diet [16]. In a study by Molina-Montez et al. in 16 European countries as well as Sidor and Rzymski, Poland is not the best, especially in terms of fruit and vegetable consumption [28,29,30]. Only in the group of people over 50, no significant changes in eating habits were observed during the COVID-19 pandemic [31].

The aim of the research was to assess the changes in physical activity, time spent in front of the monitor as well as body mass and composition, during the SARS-CoV-2 pandemic, from the implementation of distance learning (March 2020) to the loosening of restrictions introducing hybrid learning (February 2021)

## 2. Materials and Methods

The study analyzed data from 120 people aged 19.8 (±0.8). The mean height in the group was 173.2 (±9.2) cm, mean body mass 66.1(±12.8), and mean BMI 19.2 (±5.9) kg/m^2^. Groups were divided based on sex into group I (*n* = 72) women and group II (*n* = 48) men. All of the participants were students of a sports-medical university, who were attending the same year and field of study. They participated in the same types of classes, in both remote and hybrid forms. On the basis of the questionnaire, it was found that, during the period of lockdown, 72% of the participants lived in their family house, 9% in a student dormitory, 7% in a rented student flat, 2% in their own flat, and 10% changed their residence more than once in the 6-month period.

### 2.1. First Stage—Body Composition Test

The study was conducted in two stages. The first stage, December 2019, consisted of an assessment of the mass and segmental composition of the body using the electrical bioimpedance (BIA) with the use of TANITA’s 8-electrode body composition analyzer.

### 2.2. Second Stage—Study of Lifestyle Changes, Physical Activity, Questionnaire

In the second stage, February 2021, the researchers conducted a follow-up examination of body mass and composition, as well as an assessment of physical activity in relation to implemented lockdown restrictions, and incidence of SARS-CoV-2 or the appearance of symptoms accompanying the infection. The participants completed questionnaires consisting of 24 questions (12 closed and 12 open-ended) regarding the infection caused by coronavirus SARS-CoV-2, forms of physical activities undertaken, and time spent in front of a screen taking into account weekly differences (Monday–Friday period of teaching; Saturday–Sunday period free from classes). The questions were divided into 3 thematic parts. The first part concerned the development of COVID-19—were the subjects diagnosed, how hard they were, how long they were sick; quarantine, trips abroad during the pandemic (mobility during this period). The second part of the questions concerned the occurrence of symptoms of long-COVID-19 syndrome in the last 6 months. The third part of the questions (both closed and open) concerned time spent on physical activity, time spent in front of the computer, competing sports or forms of physical activity chosen by the respondent in three periods of research—before March 2020 (before the COVID-19 pandemic), lockdown period (March 2020–February 2021), after February 2021.

### 2.3. Occurrence of Specific Symptoms of Long-COVID-19 Syndrome, Questionnaire

Nonspecific symptoms of long-COVID-19 syndrome were used to assess complications related to the pandemic. According to WHO guidelines, symptoms included previously unprecedented symptoms of the respiratory system (cough, chronic fatigue, shortness of breath), cardiovascular (chest pain, palpitations), nervous system (syncope, headache, dizziness, temporary memory loss, cognitive disorders, severe problems with concentration, depressed mood, apathy, irritability, loss of sense of smell or taste), the musculoskeletal system (muscle weakness, muscle pain, numbness in the limbs, tingling limbs, joint pain, joint swelling), digestive system (significant loss of appetite), abdominal pain, diarrhea, vomiting, sore throat), mucocutaneous symptoms (skin rashes, skin changes on arms/legs, face swelling, dry red lips) or depression [32].

The questionnaire was also composed of questions, including the condition before the implementation of restrictions (confirmed by preliminary tests executed simultaneously with the first body mass and composition measurements), during the period of full lockdown, and at the time of the follow-up examination (February 2021—coinciding with the temporary implementation of hybrid classes in the form of student–lecturer contact).

The period between the first study (December 2019) and the second study (February 2021) is the time when the epidemic was introduced in Poland. It was related to the introduction of restrictions in March 2020 related to the introduction of the sanitary regime. The restrictions that influenced the functioning of the studied group were, first of all, the introduction of distance learning, the closure of sports centers, restaurants, cultural centers (cinemas, theaters) and the limitation of the possibility of gathering in groups. During this period, students most often lived in the family home, and the student dormitories were closed. This period was defined as distance learning. Distance learning was conducted (at the university from which the research group was recruited) from March 2020 to February 2021. During this period, all classes were conducted by the educational platform in a theoretical form without the possibility of personal contact. The students contacted the lecturer only through the educational portal—there were no activities based on physical activity or contact with the patient during this period.

In accordance with government guidelines, in February 2021 the restrictions were loosened and the possibility of conducting classes in a hybrid form was introduced. The respondents are students of a sports-medical university, and the study program requires activities in contact with the patient, therefore, according to the guidelines, they returned to practical exercises in contact. With the return of students to universities, research could be carried out. This period was defined as hybrid learning. Hybrid learning was introduced at the university from which the study group was recruited, at the end of February 2021 and consisted of dividing the classes into theoretical (still conducted remotely via the educational platform) and practical, taking into account physical activity (conducted in direct contact with the teacher at the university).

The difference between these forms (distance learning and hybrid learning) was that students left home for classes and the time spent in front of the monitor was limited.

**Figure 1:**
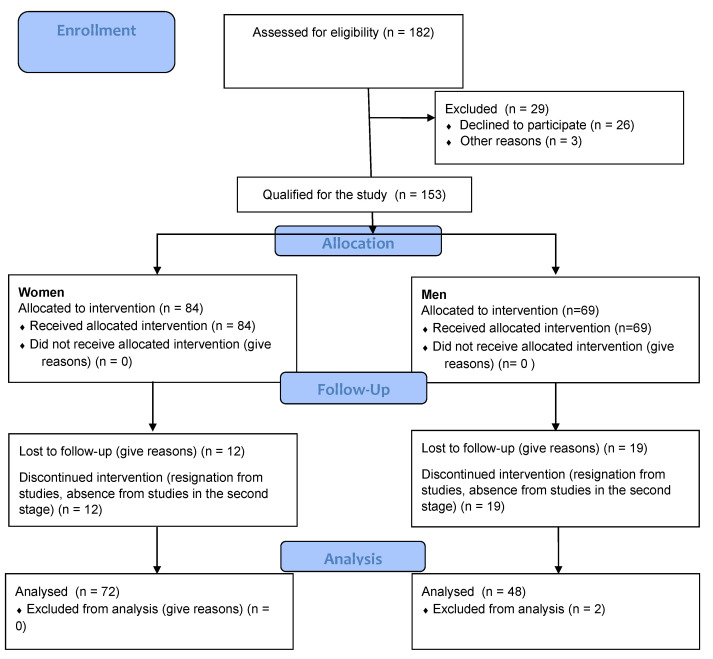
CONSORT diagram.

The study was conducted in accordance with the Declaration of Helsinki, and the research project received approval from the Senate’s Bioethical Committee at the Academy of Physical Education in Wroclaw (no. 12/2019). Signed informed consent forms were obtained from all participants. The study was conducted in accordance with the CONSORT (Consolidated Standards of Reporting Trials) statement (Figure 1).

### 2.4. Statistical Methods

The obtained results were developed with the use of the Statistica 13.3 packet. Means and standard deviations for the anthropometric data were determined. The normality of examined values was investigated using Kolmogorov–Smirnov and Lilliefors tests. For the intergroup comparison, the parametric statistics test for independent groups was used, while the comparison of the investigation results involved applying the *T*-test for dependent groups. The correlation between the anthropometric values, their changes, and the data concerning the physical activity were estimated with the use of Spearman’s rank correlation test. The significance level of *p* < 0.05 was adopted.

## 3. Results

The majority of the participants experienced self-observed COVID-19 symptoms (62%) over the last 6 months, 15% of them were infected by the virus SARS-CoV-2—among them, 7% were diagnosed via tests, and 8% based on the symptoms characteristic for this disease entity (loss of taste and smell, high temperature, headaches, muscle and joint pain. During this period, 17% of the respondents were under quarantine. The symptoms most frequently self-observed among the participants were unprecedented headaches (34%), chronic fatigue (24%), abdominal pain (25%), and musculoskeletal system symptoms such as pain and muscular weakness. In total, 13% of the participants also experienced depressive states

The comparison of symptoms categorized by sex revealed that alterations of the musculoskeletal system were more frequent among men, while 13% of women experienced nervous system symptoms (chest pains, heart palpitations, throat tightness perceived as pain, shortness of breath and dizziness, plus vomiting). Statistically significant differences were observed in regard to the incidence of chest pain—it was statistically significantly more frequent among women than men—while tingling of the extremities was statistically significantly more frequent among men than women (Table 1).

**Figure 2:**
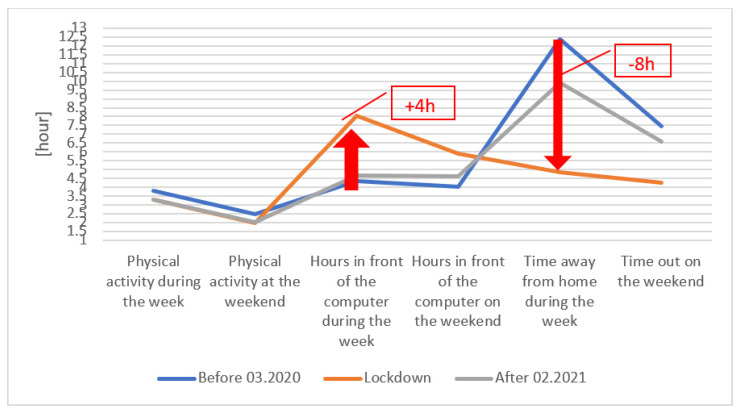
Average time spent by the surveyed students on physical activity and spent in front of a computer monitor.

In the presented research, which was conducted on a group of students, it was observed that the time devoted to physical activity had not changed significantly, however, the forms of physical activity undertaken had (Figure 2).

An average time dedicated to physical activity during the week (Mon–Fri) remained constant at around 3.5 h throughout the period of conducting the study, decreasing to around 2 h during the weekend. The types of activities undertaken by the participants changed. In the period before the start of the pandemic, the most popular forms of activity were attending fitness clubs within group or individual classes, as well as attending gyms, swimming, and playing team sports. During lockdown, the most frequent form of physical activity among women was taking individual classes at home with the use of links to online classes, while activities with the use of home gyms, jogging, cycling, or walking prevailed among men. Due to the changing form of physical activity (i.e., being performed individually), the intensity of the physical effort realized at one’s own pace changed as well. Ready sets of exercises, prepared in form of online videos, were used by 44% of female participants before the pandemic, 53% during the lockdown, and 43% in the period after February 2021. In case of men, the percentage consisted of 27%, 39%, 29%, respectively.

Significant changes in the number of hours dedicated to working in front of a computer screen and spending time outdoors were observed. During the lockdown, the average daily time that the participants spent in front of a computer was over 8 h, which is on average 4 h longer than before the pandemic. The number of hours spent outdoors, in turn, decreased by 8 h (Mon–Fri) on average. (It should be emphasized that the study was conducted on a group of adult people who were restricted solely in terms of gatherings, and not movement).

**Figure 3:**
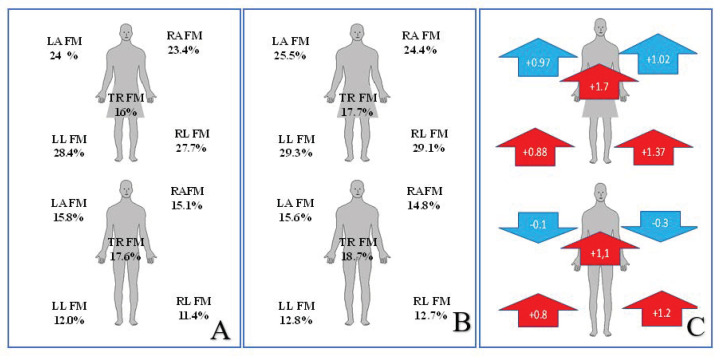
Segmental percentage of the amount of fat mass (FM) depending on gender and study, (**A**) before lockdown study, (**B**) study 02.2021, (**C**) direction of changes and their statistical significance. RL—right leg, LL—left leg, RA—right arm, LA—lest arm, TR—trunk, FM—fat mass, red color *p* < 0.005.

Fat mass was determined by examining the segmental body composition. In the group of female participants, the percentage of fat mass in all segments of the body was higher than in the male group (Figure 3A). In the study conducted in February 2021, the quantity of adipose tissue in women increased by 1.12% on average, and the biggest statistically significant changes were observed within the right lower extremity and the torso. The change in the left lower extremity is also statistically significant, however, it is the smallest of all the segments examined (Figure 3C). Attention must be paid to the fact that the body fat distribution is observably more symmetrical in the follow-up examination than in the examination before the lockdown (Figure 3B). The group of male participants presented significant changes in the mass of the adipose tissue in the lower extremities and torso, with a slight decrease in the quantity of fat mass within the upper extremities (Figure 3C).

During the examinations of body mass and composition, a statistically significant increase in the body mass and BMI of men, and the percentage fat mass of women were observed (Table 2).

In the investigated group, a statistically significant positive correlation was observed between the number of hours spent in front of a monitor/computer during a week of work/study, the increase in the percentage of adipose tissue quantity in the lower extremities (both upper and lower) and the overall fat mass of women. In the group of men, a similar dependence was observed for the lower right extremity. A negative statistically significant correlation between the change in fat mass and the time spent outdoors during the lockdown was observed in the male group (Table 3).

In the presented study, it was observed that women who did not report a loss of appetite experienced higher increases in fat mass between the first and second examination. These correlations concerned the loss of appetite and the percentage of the quantity of fat mass: overall (FM, *p* = 0.286), of both upper extremities (RA FM, *p* = 0.286; LA FM, *p* = 0.293), and of the torso (TR FM, *p* = 0.324). Men who reported experiencing a sore throat correlated with a smaller difference in the percentage quantity of fat mass in the torso area.

## 4. Discussion

The uncontrollable spread of the SARS-CoV-2 virus has changed the lifestyle to which we had been accustomed. Not only has it increased the mortality and disease incidence rates throughout the world, but it has also changed our habits, limited social contacts and forced us to restrict our mobility [16,17,18,19,20,21,22,23,24].

One of the results of implemented restrictions is a decrease in daily physical activity. As emphasized by researchers from Italy, France and Australia, Germany’s limitations caused by the pandemic mainly concerned external contacts and the need to stay at home [24]. Similar results of limiting the physical activity of students caused by the pandemic were reported by Galle et al. Researchers have observed a significant decrease in physical activity among Italian students, especially in the so-called “student life” [17]. According to the International Physical Activity Questionnaire (IPAQ), physical activity is divided into activities connected with work, commuting, domestic work, leisure and sports [7,33]. Thus, it can be noted that professional activity and commuting are important elements of physical activity. As the study results indicate, the changes reported by the participants highlighted mainly a decrease in the time spent outdoors, and the necessity to extend the time spent in a sitting position. Romero-Blanco et al., using the International Physical Activity Questionnaire (IPAQ—short form) stated that students of the Faculty of Health Sciences changed physical activity resulting from work, studies, into individual exercises. In their studies, one can observe the division of changes in physical activity depending on the occurrence of abnormalities in the body weight of the studied students [16]. Similarly, in the presented research, students changed forms of physical activity without changing the time devoted to PA. The time that the students spent in front of a computer during lockdown was two times longer than in the pre-pandemic period. A statistically significant change in the time spent in front of the computer was also observed by Majumdar et al. who examined an increase in the time spent in front of the computer by students [18]. Similar results were observed in studies on Australian students, Munasinghe et al. [20]. Even bigger differences may be observed when it comes to the possibility of leaving the house. No changes in terms of time dedicated to physical training activity among the respondents were observed. This might stem from the fact that the participants are students at a sports university and have a high level of awareness of the importance of physical activity. Similar results at the university in health sciences were obtained by Romero-Blanco et al. [16]. This may indicate a higher awareness of the respondents in the field of PA and quality of life, especially that in the studies of other authors conducted on students, no similar changes were observed [17,18,19,20,21,22,23,24]

However, the lack of change in the time dedicated to exercise, with a simultaneous change in the time spent in a sitting position (in front of the computer) and in domestic isolation, allows us to concur that academic activity (following the university curriculum) combined with active leisure may affect the preservation of a correct body mass. This is evidenced by the results obtained, which show that limiting these two aspects of everyday life could have a negative impact on the weight and body composition of the participants. The impact of the restrictions stemming from the pandemic period on human functioning was confirmed also by Tan et al., who brought to attention the change of eating habits namely that of replacing healthy nutrition with processed products which have a longer expiry date [34]. The problem of unhealthy food and the decrease in the time dedicated to exercising during the pandemic was also notified by Pietrobelli et al. in the research conducted on obese children and adolescents in Italy [35]. Additionally, the investigations of Robinson et al. have shown a change in diet and physical activity undertaken, coinciding with the pandemic restrictions, which, according to the researchers, will have long-lasting consequences [7].

It should be noted that maintaining correct muscle mass, which is a substantial part of the lean body mass, requires undertaking physical activity. Limiting one form of activity, for instance professional activity, should be compensated by increasing the intensity of physical effort outside of work. In order to maintain a healthy fat mass to lean body mass ratio, an increase in the intensity of undertaken physical activity is recommended. In the investigated group, the time devoted to physical activity has not changed, but the form of the activity has. Exercises realized in groups (fitness, team sports) were replaced by individual running, or, in the case of women, virtual exercise classes with the use of online videos. Unfortunately, it negatively affected the participants’ body composition, and increased the fat mass within the torso and lower extremities. It should be noted that the increase in fat mass among women occurred at the expense of muscle mass, as no statistical change in body mass was observed. In the case of men, the average change in body mass was 1.5 kg, while fat mass increased by 1 kg on average. Men aged 18–21 are people in a stable ontogenetic period and according to the norm should have fat mass within the limits of 8–20% of body mass [36,37,38,39]. Their change in body mass or change in fat mass is the effect of an unbalanced energy balance. The presented calculations determine how much fat mass men who have gained weight should gain in the analyzed period. If we assume that the body mass of the studied men increased by an average of 1.5 kg, then (at 20% FM) they should acquire a maximum of 300 g of fat mass and they gained an average of 1 kg. This shows a change in the structure of the body mass and a change in the ratio of fat to lean mass. If the subjects gained (with a weight increase of 1.5 kg) up to 300 g of fat mass, they would remain in the same proportions of body composition components. However, they gained three times more fat mass than they could gain 1.5 kg of body mass [27,28,29,30]. In the investigated group, for the increase of body mass to be within normal limits, fat mass should increase within the limits of 100–300 g, however, it increased by on average 1 kg. This might be indicative of a too low intensity of physical effort or incorrect diet over the period of lockdown. The influence of nutrition on the changes in body composition in the investigated group requires further research.

An alarming result of the presented research is its psychological aspect which indicates the incidence of depressive symptoms and nervous system symptoms in the studied group. One of the most dangerous symptoms resulting from the isolation are depressive states, which were self-observed by as many as 13% of examined students. Similar results of the increase in mental anxiety among especially young women were observed by researchers in the USA and UK [21,26,27]. It is worth noting that before the pandemic period, WHO estimated that 5–6% of the world’s adult population suffered from depression, while studies conducted on groups of medical students (doctors, nurses, physiotherapists) showed that, on average, 15–20% experienced reduced well-being, and 5% suffered from severely depressed mood and depressive states. No correlation was observed between the mental condition and the changes in body composition. This aspect requires further research on a larger group [40,41,42,43].

The substantial changes in fat mass observed in the presented study are alarming, as the examined group is at a relatively stable stage of ontogenetic development, and additionally, is aware of the importance of physical activity for the quality of life.

The presented results, similar to the research of other authors [7,8,9,15,16,17,18,19,20,21,22,23,24,33,34], confirm that post-pandemic complications will affect not only people who tested positive for SARS-Cov-2, but also entire populations.

The authors who conducted research on the incidence of post-COVID-19 complications observed the lack of criteria for the occurrence of symptoms of long-COVID-19 syndrome. In the publications to date, only non-specific symptoms appear, the main qualifying element of which is an earlier contracting of SARS-CoV-2 virus. However, in the studied age group, the infection could be asymptomatic, therefore, it is difficult to diagnose long-COVID-19 syndrome, and thus to fully describe the impact of a pandemic on young people another study should be carried out on a larger group.

## 5. Conclusions

In the studied group, there was a change in the forms of physical activity from strength and group activity to endurance activity (running forms, cycling) and individual activity using links to exercises available on the Internet. The subjects showed a statistically significant increase in body fat, regardless of gender, and in the upper limbs in men

## Figures and Tables

**Table 1:** Symptoms of infection that occurred in the respondents during the last 6 months before the control examination.

		Women (%)	Men (%)	Pearson Chi-Square Test
There were symptoms (at least one)		59	63	NS
	chronic fatigue	24	23	NS
	headaches	38	20	NS
	dizziness	18	11	NS
Respiratory disorders	throat tightness perceived as pain	12	9	NS
	chest pain	12	0	0.033
	shortness of breath	2	3	NS
	breathlessness	4	11	NS
Musculoskeletal system symptoms	muscle pain	10	17	NS
	joint pain	16	6	NS
	numbness in the limbs	8	8.5	NS
	tingling in the limbs	6	20	0.048
	joint swelling	0	3	NS
	muscle weakness	4	17	NS
	heart palpitations	4	0	NS
	skin rashes	14	3	NS
	skin changes on the hands/feet	4	0	NS
Mental symptoms	depressive states	10	11	NS
	cognitive impairment	4	3	NS
Digestive system disorders	abdominal pain	26	20	NS
	diarrhea	0	3	NS
	plus vomiting	6	3	NS
	loss of taste	4	6	NS
	loss of smell	2	6	NS
	significant loss of appetite	4	3	NS

**Table 2:** Anthropometric measurements of the studied women and men.

	Women			Men		
	12.2019	02.2021	*p*	12.2019	02.2021	*p*
Body mass (kg)	57.5 ± 8.2	57.8 ± 9.5	NS	78.2 ± 9.2	79.7 ± 9.4	0.049
BMI (k/m^2^)	20.4 ± 2.3	20.6 ± 2.8	NS	23.3 ± 2.6	23.8 ± 2.8	0.018
Fat mass (FM) (%)	21.1 ± 4.8	22.5 ± 5.3	0.000	15.4 ± 5.7	16.3 ± 5.8	NS

**Table 3:** Correlation of time spent in front of the monitor and away from home and the change in body fat in the study group.

		FM 2020–2021	RL FM 2020–2021	LL FM 2020–2021	RA FM 2020–2021	LA M 2020–2021	TR FM 2020–2021
Women	How many hours a day did you spend in front of the computer on Mon–Fri (on average, taking into account the time spent on remote teaching)—lockdown	0.335	0.098	0.469	0.266	0.299	0.184
Men	How many hours a day do you spend in front of the computer—in weekend lockdown	0.163	0.394	0.075	0.152	0.170	0.084
	How much time do you spend outside your home on average (Mon–Fri)—lockdown	−0.385	−0.292	−0.449	−0.270	−0.312	−0.280
	How much time do you spend outside your home on average—in weekend lockdown	−0.244	−0.390	−0.338	−0.047	−0.130	−0.137

## Data Availability

The research results presented are part of a large ongoing study which has not yet been completed. If you are interested in specific data, please contact the first author—Agnieszka Chwałczyńska (agnieszka.chwalczynska@awf.woc.pl).
